# A lower maximum bite force is a risk factor for developing cardiovascular disease: the Suita study

**DOI:** 10.1038/s41598-021-87252-5

**Published:** 2021-04-07

**Authors:** Sakae Hashimoto, Takayuki Kosaka, Michikazu Nakai, Momoyo Kida, Shuri Fushida, Yoshihiro Kokubo, Makoto Watanabe, Aya Higashiyama, Kazunori Ikebe, Takahiro Ono, Yoshihiro Miyamoto

**Affiliations:** 1 Department of Prosthodontics, Gerodontology and Oral Rehabilitation, Osaka University Graduate School of Dentistry, 1-8 Yamadaoka, Suita, Osaka 565-0871 Japan; 2 Open Innovation Center, National Cerebral and Cardiovascular Center, 6-1 Kishibe-Shimmachi, Suita, Osaka 564-8565 Japan; 3 Department of Preventive Cardiology, National Cerebral and Cardiovascular Center, 6-1 Kishibe-Shimmachi, Suita, Osaka 564-8565 Japan; 4 Department of Hygiene, Wakayama Medical University, 811-1, Kimiidera, Wakayama, 641-8509 Japan; 5 Division of Comprehensive Prosthodontics, Niigata University Graduate School of Medical and Dental Sciences, 2-5274, Gakkocho-dori, Niigata, 951-8514 Japan

**Keywords:** Cardiology, Medical research, Risk factors

## Abstract

Decreases in masticatory function are believed to be related to the development of cardiovascular disease (CVD) through inappropriate nutritional intake. This study focused on maximum bite force (MBF), which is an objective, quantitative index of masticatory function, and its association with the development of CVD (stroke and coronary heart disease) was investigated. The subjects were 1547 participants of the Suita study with no history of CVD who underwent medical and dental health examinations between June 2008 and June 2013. In addition to undergoing a basic physical examination at baseline, their MBF was measured. They subsequently underwent follow-up surveys for the development of CVD (mean follow-up, 3.5 years). The association between baseline MBF and the development of CVD was investigated by multivariate adjustment using a Cox proportional hazards model. CVD developed in 32 subjects during follow-up. The trend test showed a significant association between baseline MBF and CVD in a model that combined men and women. When analysed by sex, the trend test found a significant association between baseline MBF and CVD in women. Low MBF, which is an objective and quantitative index of masticatory function, may be a risk factor for the development of CVD.

## Introduction

Cardiovascular disease (CVD) is a leading cause of death^1^. Even if survival can be extended during the acute phase, repeated exacerbations and recurrences diminish the quality of life (QOL), and may, in the worst case, result in death. The prevention of CVD thus averts decreased QOL and is an extremely important issue for maintaining healthy living.

Previous studies have found that periodontitis^2,3^ and the number of teeth^4,5^, which are indices of oral health, are associated with CVD. CVD is caused by two pathways: via chronic inflammation due to periodontitis; and by inappropriate nutritional intake as a result of reduced masticatory function due to tooth loss^6^. The effect of inappropriate dietary intake has been described in studies of the association between the incidence of CVD and the subjective assessment of masticatory function in terms of chewable foods^7,8^. However, no previous research has investigated the association between masticatory function assessed in objective and quantitative terms and the development of CVD.

Maximum bite force (MBF) is an objective, quantitative index of masticatory function, and it is measured in both everyday clinical practice and research studies. Studies to date have identified factors such as not only the number of teeth^9^ and periodontal disease^10^, but also physical performance^11^ as factors related to decreased MBF. Therefore, it was reported that MBF was strongly associated with masticatory ability^12^. On the other hand, low MBF has been reported to reduce the intake of minerals, vitamins, and dietary fibre^13^.

In the present study, we hypothesized that low MBF is associated with the incidence of CVD, and a 3.5-year follow-up study of a general urban population was performed.

## Methods

### Population

The Suita study, a cohort study of CVD of urban residents, was established in 1989. The details of this study have been described elsewhere^14^. Oral examinations of consenting participants have been conducted since June 2008.

**Figure 1:**
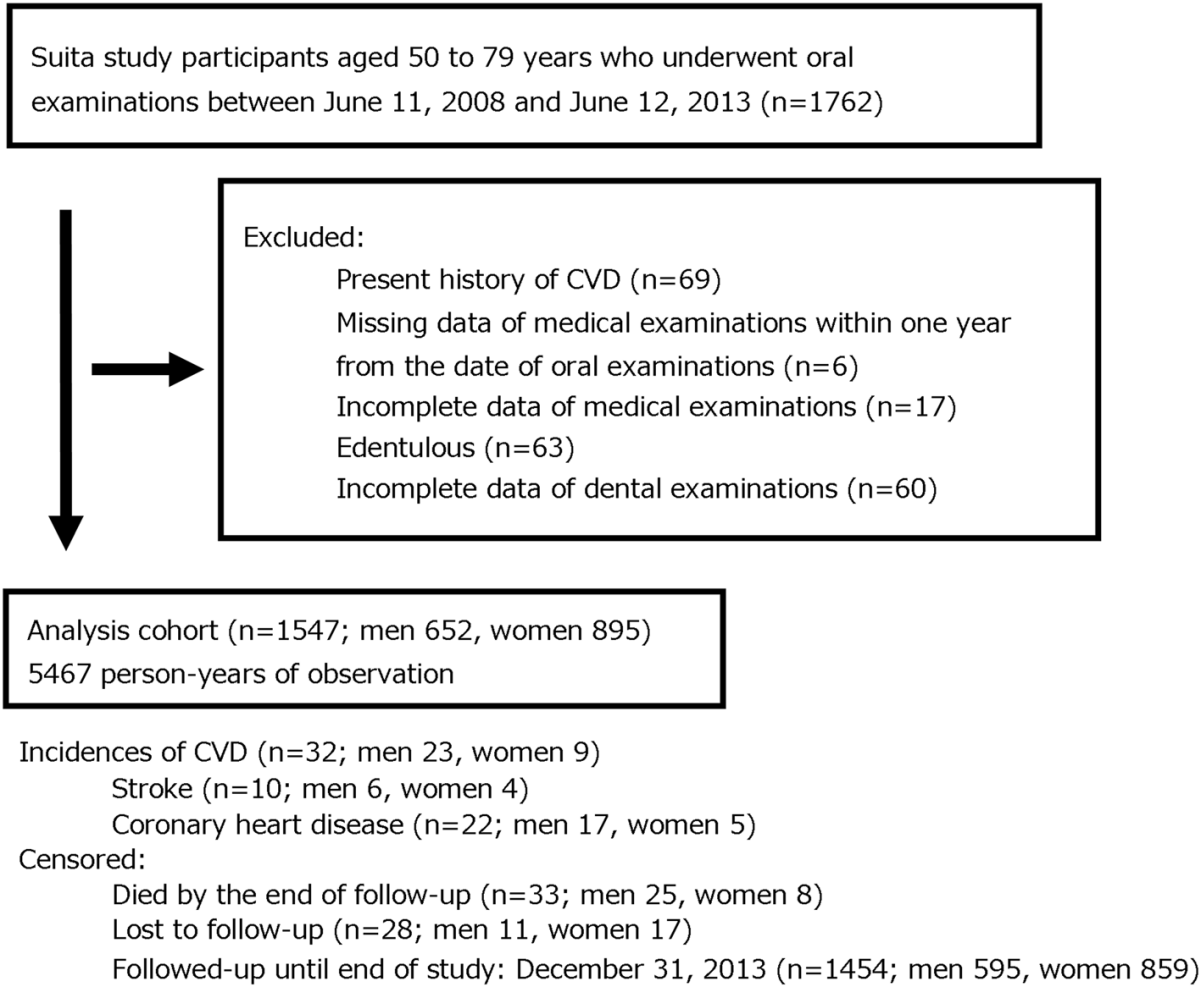
Flow chart of study participants. CVD, cardiovascular disease.

For these participants, the baseline of the present study was set at medical and oral examinations held from June 2008 to June 2013. During this period, 1762 participants attended the baseline survey and were followed until the end of 2013. Of these, 215 were excluded for the following reasons: history of CVD (n = 69), no medical examination data from within one year of oral examination available (n = 6), incomplete medical examination data (n = 17), fully edentulous (n = 63), or incomplete oral examination data or MBF measurements (n = 60) (Fig. 1). The final analysis cohort included 1547 subjects (652 men and 895 women; mean age at baseline 66.8 years for men and 65.6 years for women).

This cohort study was approved by the ethics committee of the National Cerebral and Cardiovascular Center (M25-032–2). Informed consent was obtained from all participants after receiving a full written and oral explanation of the study purpose and methods in advance were surveyed. All methods were performed in accordance with the relevant guidelines and regulations.

### Baseline examination

#### Medical examination

Height and weight were measured and used to calculate the body mass index (BMI). Blood pressure was measured twice with a standard auto sphygmomanometer (BP103i2, Omron, Kyoto, Japan). The mean values of systolic and diastolic blood pressures from the second and third measurements were used for analysis. Blood for blood tests was drawn after the subjects had been fasting for at least 12 h.

Hypertension was defined as systolic blood pressure ≥ 140 mmHg, diastolic blood pressure ≥ 90 mmHg^15^ or the use of antihypertensive medication. Non-high-density lipoprotein (non-HDL) cholesterol was calculated by subtracting HDL cholesterol from serum total cholesterol. The National Glycohemoglobin Standardization Program Method was used for haemoglobin A1c (HbA1c)^16^. Diabetes mellitus was defined as HbA1c (NGSP value) ≥ 6.5%^17^ or the use of antidiabetic medication. Dyslipidaemia was defined as non-HDL cholesterol ≥ 170 mg/dL^18^ or the use of antilipidaemic medication.

#### Lifestyle variables

A nurse went through a medical questionnaire with the participants about their smoking, drinking, physical activity, and medical history. Smoking and drinking were classified as either current or not current. Physical activity was classified as active or inactive depending on whether the participant engaged in activity equivalent to walking for at least 60 min daily.

#### Oral examination

The oral examination was conducted with the participant lying supine on a bed under sufficiently bright artificial lighting, and it consisted of checking the number of remaining teeth, the number of functional teeth, and the health of periodontal tissue.

##### Number of functional teeth

The number of functional teeth was counted as the total number of both natural and treated teeth contributing to masticatory function, including pontics in fixed partial dentures and implant-supported dental prostheses, but excluding wisdom teeth.

##### Periodontal tissue examination

The health of periodontal tissue was evaluated in terms of the Community Periodontal Index (CPI)^19^. A total of ten designated teeth were examined, comprising the upper and lower bilateral first and second molars, the upper right central incisor, and the lower left central incisor, and if one of these incisors was missing, then the corresponding tooth on the opposite side was examined instead. Five dentists who underwent advance calibration used a CPI probe (YDM, Tokyo, Japan) to examine six points around each tooth for periodontal pockets according to the following conditions, and they recorded the highest code for each tooth. The CPI codes were as follows: Code 0, no sign of gingival inflammation; Code 1, bleeding on probing; Code 2, calculus deposition (including detection of pockets up to 4 mm deep by probing); Code 3, periodontal pockets of depth ≥ 4 mm but < 6 mm; and Code 4, periodontal pockets of depth ≥ 6 mm. Cohen’s κ for agreement between the results of periodontal tissue examinations by the five dentists was 0.78. In this study, teeth with a CPI code of 3 or 4 were classed as having periodontitis, and the subjects were divided into those without periodontitis (CPI 0–2) and those with periodontitis (CPI 3–4).

#### MBF measurement

MBF was measured with the subject seated. A 98-μm-thick pressure-sensitive sheet (Dental Prescale 50H type R, GC, Tokyo, Japan) was used for MBF measurement. With the subjects seated, head position was adjusted so that the Frankfurt plane was parallel to the floor. The sheet was inserted between the upper and lower teeth, and the patient was instructed to bite down gently, after which they were instructed to bite on it as hard as they could for 3 s^20^. This sheet consists of two pieces of polyethylene terephthalate film enclosing countless pigment-containing microcapsules between them. When the subject bites down on the sheet, the microcapsules within it rupture, releasing the pigment within them and staining the sheet red. The concentration of the stain varies according to the strength of the force applied. MBF was calculated by scanning the area and the concentration of staining on the sheet with a special image scanner (OCCLUZER FPD-709, GC). This method reflects the bite strength exerted by the entire dentition near the maximal intercuspal position^21^. This measurement method requires no special equipment during measurements other than the sheet, is simple and quick to perform, and has been reported to be both highly accurate^22^ and highly reproducible^9^ when conducted following the measurement procedure.

MBF was measured twice, and the mean value was used as the subject’s measured value. Participants who used removable dentures wore them during measurements^23^.

### Confirmation of stroke and coronary heart disease and endpoint determination

The confirmation of stroke and coronary heart disease (CHD) in the Suita Study has been described elsewhere^14^. In brief, medical records were reviewed by registered hospital physicians or research physicians who were blinded to the baseline data.

Strokes were defined in accordance with the U.S. National Survey of Stroke criteria^24^. Definitive diagnoses for each stroke subtype (cerebral infarction, intracerebral haemorrhage, and subarachnoid haemorrhage) were established based on examination of computed tomography scans, magnetic resonance images, or autopsies.

Definite and probable myocardial infarctions were defined according to the criteria set out by the Monitoring Trends and Determinants of Cardiovascular Disease project (MONICA)^25^. In addition, CHD consisted of coronary angioplasty, coronary artery bypass surgery, and sudden cardiac death within 24 h. In this study, CVD was defined as stroke and CHD.

To detect stroke and CHD occurrences, the subjects attended the National Cerebral and Cardiovascular Center every two years for a general health examination. All subjects were also asked to complete a questionnaire either by telephone or mail each year. In addition to completing the surveillance for fatal strokes and CHDs, a systematic search of death certificates was conducted. All the data (health examinations, questionnaire surveys, and death certificates) were checked against medical records to confirm the occurrence of CVD. Possible strokes and CHDs were identified using data from the results of (1) health examinations and questionnaires on the history of stroke or CHD for subjects who did not consent to the medical records survey and (2) death certificates of suspected stroke or CHD cases when the occurrence of CVD had not been recorded.

The endpoints of this study were the date of the first stroke or CHD, date of death, date of moving out of Suita City, or December 31, 2013 (censored date).

### Statistical analysis

Baseline MBF was divided into quintiles separately for men and women, and a sex-stratified analysis was conducted. The baseline characteristics of study subjects in the different groups were compared using analysis of variance or the χ^2^ test. Multivariate-adjusted hazard ratios (HRs) of MBF for CVD were calculated using a proportional hazards model adjusted for age, sex, number of functional teeth, periodontitis, BMI, smoking (current smoker or non-smoker), drinking (current drinker or non-drinker), physical activity (engaging in activity equivalent to walking for 60 min per day), hypertension, dyslipidaemia, and diabetes mellitus. Analyses, which were conducted after a log–log plot was prepared and the proportional hazards property was verified, showed that there was no interaction between sex and MBF for the development for CVD. The Cochran-Armitage Test was used.

IBM SPSS Statistics 25 (SPSS Japan Inc., IBM, Tokyo, Japan) was used for statistical analysis, with the level of significance set at 5%.

## Results

Table 1 shows the baseline characteristics of the study subjects separately for men and women. MBF was significantly higher in men than in women (*p* < 0.001).

**Table 1:** Subjects’ baseline characteristics.

	All	Men	Women	*p* Value
Number	1547	652	895	
Person-years	5467	2299	3167	
MBF, N	504 ± 283	552 ± 307	469 ± 259	< 0.001
Age, y	66.1 ± 7.9	66.8 ± 7.9	65.6 ± 7.9	0.003
BMI, kg/m^2^	22.7 ± 3.1	23.5 ± 2.9	22.1 ± 3.2	< 0.001
SBP, mmHg	127.6 ± 19.7	129.9 ± 18.6	125.9 ± 20.3	< 0.001
DBP, mmHg	77.6 ± 11.3	80.3 ± 10.8	75.7 ± 11.2	< 0.001
Non-HDL cholesterol, mg/dL	149.5 ± 32.1	142.5 ± 29.8	154.6 ± 32.7	< 0.001
HbA1c, %	5.8 ± 0.6	5.9 ± 0.8	5.8 ± 0.5	0.005
Hypertension, %	45.6	52.3	40.8	< 0.001
Hyperlipidaemia, %	48.4	41.3	53.5	< 0.001
Diabetes mellitus, %	12.0	17.6	7.8	< 0.001
Smoking status, %	10.7	19.3	4.4	< 0.001
Drinking status, %	45.8	69.3	28.7	< 0.001
Physical activity, %	39.3	41.3	37.9	0.179
Number of functional teeth	24.1 ± 5.9	23.6 ± 6.4	24.5 ± 5.5	0.003
Periodontitis, %	49.3	56.9	43.7	< 0.001

Data are frequencies or means ± SD.

MBF, maximum bite force; BMI, body mass index; SBP, systolic pressure; DBP, diastolic blood pressure; HDL, high-density lipoprotein-cholesterol; Physical activity, ≥ 60 min walking per day.

Analysis of variance was used for comparisons of multiple group means, and the Chi-squared test was used to compare frequencies.

Tables 2 and 3 show the baseline characteristics of the study subjects by MBF quintiles separately for men and women. In both men and women, groups with higher MBF were significantly younger and had significantly more functional teeth. In men, groups with higher MBF included a significantly larger proportion of drinkers, but this association was not evident in women.

**Table 2:** Subjects’ baseline characteristics by MBF quintiles in men.

	All	Q1	Q2	Q3	Q4	Q5	*p* Value
Number	652	130	131	130	132	129	
Person-years	2299	474.3	459.8	479.5	445.6	474.3	
MBF, N	552 ± 307	171 ± 75	367 ± 51	527 ± 39	687 ± 53	1010 ± 229	< 0.001
Age, y	66.8 ± 7.9	69.4 ± 7.7	68.0 ± 8.0	65.1 ± 7.5	66.6 ± 8.2	64.8 ± 7.5	< 0.001
BMI, kg/m^2^	23.5 ± 2.9	23.4 ± 3.1	23.5 ± 3.2	23.2 ± 2.4	23.6 ± 2.8	23.6 ± 2.9	0.789
SBP, mmHg	129.9 ± 18.6	128.9 ± 19.9	132.3 ± 18.0	125.9 ± 17.4	132.9 ± 18.1	129.5 ± 18.7	0.016
DBP, mmHg	80.3 ± 10.8	78.7 ± 10.6	80.5 ± 10.2	79.1 ± 10.8	82.6 ± 11.4	80.8 ± 10.6	0.031
Non-HDL cholesterol, mg/dL	142.5 ± 29.8	140.0 ± 30.2	143.7 ± 32.3	144.6 ± 29.9	144.6 ± 25.3	139.5 ± 30.9	0.440
HbA1c, %	5.9 ± 0.8	6.0 ± 0.9	5.9 ± 0.9	5.8 ± 0.7	5.8 ± 0.6	5.9 ± 0.8	0.478
Hypertension, %	52.3	53.8	55.0	43.8	58.3	50.4	0.175
Hyperlipidaemia, %	41.3	36.9	42.0	38.5	43.9	45.0	0.631
Diabetes mellitus, %	17.6	23.8	22.6	12.2	18.9	14.7	0.061
Smoking status, %	19.3	20.8	21.4	19.2	22.0	13.2	0.374
Drinking status, %	69.3	56.2	67.9	70.0	72.0	80.6	0.001
Physical activity, %	41.3	39.2	42.7	40.8	37.9	45.7	0.728
Number of functional teeth	23.6 ± 6.4	16.6 ± 8.3	22.6 ± 6.2	25.2 ± 4.2	26.4 ± 2.8	27.1 ± 1.5	< 0.001
Periodontitis, %	56.9	56.2	56.5	58.5	52.3	61.2	0.679

Data are frequencies or means ± SD.

MBF, maximum bite force; BMI, body mass index; SBP, systolic pressure; DBP, diastolic blood pressure; HDL, high-density lipoprotein-cholesterol; Physical activity, ≥ 60 min walking per day.

Analysis of variance was used for comparisons of multiple group means, and the Chi-squared test was used to compare frequencies.

**Table 3:** Subjects’ baseline characteristics by MBF quintiles in women.

	All	Q1	Q2	Q3	Q4	Q5	*p* Value
Number	895	179	179	179	179	179	
Person-years	3167	644.9	649.7	627.6	635.1	609.9	
MBF, N	469 ± 259	153 ± 58	312 ± 37	441 ± 36	574 ± 45	863 ± 193	< 0.001
Age, y	65.6 ± 7.9	66.9 ± 8.1	66.4 ± 7.8	64.7 ± 7.9	65.4 ± 7.8	64.5 ± 7.5	0.015
BMI, kg/m^2^	22.1 ± 3.2	22.0 ± 3.1	22.6 ± 3.3	21.6 ± 3.2	22.3 ± 3.2	22.1 ± 3.2	0.082
SBP, mmHg	125.9 ± 20.3	128.0 ± 20.7	126.2 ± 19.8	122.6 ± 21.2	127.2 ± 19.8	125.4 ± 19.7	0.110
DBP, mmHg	75.7 ± 11.2	75.7 ± 11.0	75.8 ± 10.4	74.3 ± 11.7	76.3 ± 11.6	76.2 ± 11.2	0.424
Non-HDL cholesterol, mg/dL	154.6 ± 32.7	152.1 ± 34.6	155.7 ± 29.8	155.3 ± 30.8	155.7 ± 35.6	154.1 ± 32.7	0.820
HbA1c, %	5.8 ± 0.5	5.8 ± 0.6	5.8 ± 0.5	5.8 ± 0.4	5.8 ± 0.6	5.8 ± 0.5	0.700
Hypertension, %	40.8	42.5	43.0	33.5	45.3	39.7	0.195
Hyperlipidaemia, %	53.5	50.3	59.8	49.7	52.5	55.3	0.296
Diabetes mellitus, %	7.8	8.4	6.1	5.6	10.1	8.9	0.468
Smoking status, %	4.4	4.5	5.0	6.1	2.8	3.4	0.548
Drinking status, %	28.7	27.4	24.6	30.7	24.0	36.9	0.045
Physical activity, %	37.9	38.0	35.2	34.6	44.1	37.4	0.362
Number of functional teeth	24.5 ± 5.5	20.0 ± 7.8	23.7 ± 5.3	25.8 ± 3.7	26.0 ± 3.7	27.0 ± 2.0	< 0.001
Periodontitis, %	43.7	43.6	46.9	40.8	47.5	39.7	0.462

Data are frequencies or means ± SD.

MBF, maximum bite force; BMI, body mass index; SBP, systolic pressure; DBP, diastolic blood pressure; HDL, high-density lipoprotein-cholesterol; Physical activity, ≥ 60 min walking per day.

Analysis of variance was used for comparisons of multiple group means, and the Chi-squared test was used to compare frequencies.

The total number of person-years covered by the investigation was 5467 person-years (2299 person-years for men and 3167 person-years for women), and mean follow-up was 3.5 years. New CVD occurred in 32 subjects during follow-up (10 cases of stroke and 22 of myocardial infarction).

Table 4 shows MBF stratified by quintiles, the number of cases of CVD in each quintile, and the multivariate-adjusted HRs at each level. Since there were fewer cases of CVD in women, they were stratified into three levels by combining the 2nd and 3rd quintiles and the 4th and 5th quintiles.

**Table 4:** Multivariable-adjusted hazard ratios and 95% confidence intervals for CVD by MBF quintile.

MBF quintile	MBF range, N	n	CVD events, n	HR^a^	95% CI	HR^b^	95% CI
*Men and women combined (n = 1547)*							
Q1		309	12	1.00		1.00	
Q2		310	8	0.74	0.30, 1.81	0.63	0.24, 1.66
Q3		309	6	0.61	0.23, 1.65	0.59	0.20, 1.74
Q4		311	4	0.41	0.13, 1.27	0.35	0.10, 1.24
Q5		308	2	0.23	0.05, 1.03	0.19	0.04, 0.96
				P for trend	0.03	P for trend	0.03
*Men (n = 652)*							
Q1	< 288.4	130	7	1.00		1.00	
Q2	288.4–459.5	131	6	1.03	0.34, 3.08	1.07	0.32, 3.55
Q3	459.6–593.4	130	5	0.94	0.29, 3.06	1.25	0.33, 4.74
Q4	593.5–776.3	132	4	0.76	0.22, 2.62	0.88	0.21, 3.75
Q5	> 776.3	129	1	0.22	0.03, 1.81	0.25	0.03, 2.45
				P for trend	0.16	P for trend	0.28
*Women (n = 895)*							
Q1	< 240.4	179	5	1.00		1.00	
Q2 + Q3^b^	240.4–501.4	368	3	0.31	0.07, 1.30	0.25	0.06, 1.14
Q4 + Q5^b^	> 501.5	368	1	0.11	0.01, 0.95	0.11	0.01, 1.00
				*p* for trend	0.03	*p* for trend	0.03

MBF, maximum bite force; CVD, cardiovascular disease; HR, hazard ratio; C.I., confidence interval.

Men and women combined model HR also adjusted for sex.

^a^Adjusted for age.

^b^Adjusted for age, number of functional teeth, periodontitis, BMI, smoking status, drinking status, physical activity, hypertension, hyperlipidaemia, and diabetes mellitus.

When MBF was analysed as a continuous variable, the incidence of CVD was higher in both men and women with low baseline MBF. However, the HRs were not significant for men, but they were significant for women (men: age-adjusted HR 0.90, 95% CI 0.77–1.05; multivariate-adjusted HR 0.91, 95% CI 0.76–1.09; women: age-adjusted HR 0.60, 95% CI 0.40–0.91; multivariate-adjusted HR 0.54, 95% CI 0.33–0.88).

When MBF was analysed by quintiles, taking baseline MBF in the 1st quintile as the reference value, the HR was lowest in the 5th quintile. However, the HRs were not significant for men, but they were significant for women (men: age-adjusted HR 0.22, 95% CI 0.03–1.81; multivariate-adjusted HR 0.25, 95% CI 0.03–2.45; women: age-adjusted HR 0.11, 95% CI 0.01–0.95; multivariate-adjusted HR 0.11, 95% CI 0.01–1.00).

The trend test found a significant association between baseline MBF and CVD in a model that combined the quintiles of men and women (*p* = 0.03). When analysed by sex, the trend test found no significant association in men, but there was a significant association between baseline MBF and CVD in women (*p* = 0.03).

## Discussion

This study investigated the impact of MBF, which is an objective, quantitative index of masticatory function, on the development of CVD in a 3.5-year cohort study of a random sample of a general urban population. It was found that individuals with low MBF are at higher risk of future CVD, suggesting that MBF may be considered a new risk marker for the development of CVD.

The following pathway is one potential mechanism whereby diminished masticatory function may affect the development of CVD. As masticatory function is reduced by age and tooth loss, the choice of foods that individuals can eat becomes more restricted. In practical terms, they avoid hard foods that are difficult to chew and favour those that are softer and easier to masticate, ingesting less vegetables and vitamins as a result^26,27^. Low MBF has been reported to reduce fruit and vegetable intake, leading to reduced intake of antioxidant vitamins and dietary fibre^13^. Other studies have found that ingesting antioxidant vitamins and dietary fibre by eating vegetables decreases the incidence of CVD^28,29^. Thus, one explanation is that diminished masticatory function leads to inappropriate nutritional intake, resulting in a lower intake of antioxidant vitamins and dietary fibre, which leads to the development of CVD. However, no previous study has investigated the association between an objective, quantitative index of masticatory function and the development of CVD, and the present study is the first to provide findings that support the pathway described above.

An association between the development of CVD and the number of teeth as an index of oral health has also been reported^4,5^, but the present study identified a significant association between MBF and the development of CVD even adjusted for the number of functional teeth. Compared with morphological assessments based on tooth count, the present method of measuring MBF as an objective, quantitative evaluation of actual masticatory function may provide a new approach to the pathway whereby diminished masticatory function leads to the development of CVD.

In the present study, although MBF was significantly correlated with the development of CVD overall in women, this correlation was not significant in men. One reason for this sex difference in the association between MBF and the development of CVD may be that a larger proportion of men have systemic risk factors for the development of CVD than women. In fact, an examination of the baseline characteristics of the study subjects showed that the CVD-associated factors of history of hypertension, diabetes mellitus, smoking, and drinking were all more common in men than in women (Table 1). It is possible that these factors may have had a stronger effect in men, resulting in no significant association being observed between MBF and the development of CVD.

However, this study had a number of limitations. The Suita Study is a cohort study that was launched in 1989, but the dental research team only joined the study in 2008. Therefore, only a short time had elapsed since the baseline examination period designated in this study, meaning that only a limited number of cases of CVD occurred. The particularly low number of cases in women meant that the analytical procedure for the quintile model had to be adapted by combining the 2nd and 3rd quintiles and the 4th and 5th quintiles. The second limitation is that, although MBF, which is an objective, quantitative index of masticatory function, was used, it was not possible to assess subjects’ actual nutritional intake in the baseline examinations. Although the present results are thus supported by those from many other previous studies, the pathway whereby low MBF affects the development of CVD via nutritional intake remains unknown and purely in the realm of speculation.

## Conclusions

In conclusion, in a model that combined men and women, there was a significant association between low MBF and the development of CVD even after adjusting for sex as a confounding factor. The results of this study also suggest that individuals with low MBF have a higher risk of CVD, particularly women.

Evaluating MBF as an index of masticatory function and providing appropriate dental intervention based on the results in order to improve and maintain MBF may contribute to the prevention of CVD.
